# Highly sensitive sensing of CO and HF gases by monolayer CuCl

**DOI:** 10.1039/d4ra01519c

**Published:** 2024-05-21

**Authors:** Shamiala Pervaiz, M. Usman Saeed, Sehrish Khan, Bisma Asghar, Y. Saeed, Hosam O. Elansary, A. U. R. Bacha

**Affiliations:** a Department of Physics, Abbottabad University of Science and Technology Abbottabad KPK Pakistan yasir.saeed@kaust.edu.sa yasirsaeedphy@aust.edu.pk +(92)-3454041865; b Prince Sultan Bin Abdulaziz International Prize for Water Chair, Prince Sultan Institute for Environmental, Water and Desert Research, King Saud University Riyadh 11451 Saudi Arabia; c Department of Plant Production, College of Food & Agriculture Sciences, King Saud University Riyadh 11451 Saudi Arabia; d State Key Laboratory of Urban Water Resource and Environment, Shenzhen Key Laboratory of Organic Pollution Prevention and Control, School of Civil and Environmental Engineering, Harbin Institute of Technology Shenzhen Shenzhen 518055 P. R. China

## Abstract

Using a first-principles approach, the adsorption characteristics of CO and HF on a CuCl monolayer (ML) are studied with Grimme-scheme DFT-D2 for accurate description of the long-range (van der Waals) interactions. According to our study, CO gas molecules undergo chemisorption and HF gas molecules show a physisorption phenomenon on the CuCl monolayer. The adsorption energy for CO is −1.80 eV, which is quite a large negative value compared to that on other previously studied substrates, like InN (−0.223 eV), phosphorene (0.325 eV), Janus Te_2_Se (−0.171 eV), graphene (P-graphene, −0.12 eV, B-graphene, −0.14 eV, N-graphene, −0.1 eV) and monolayer ZnS (−0.96 eV), as well as pristine hBN (0.21 eV) and Ti-doped hBN (1.66 eV). Meanwhile, for HF, the adsorption energy value is −0.31 eV (greater than that of Ti-doped hBN, 0.27 eV). For CO, the large value of the diffusion energy barrier (DEB = 1.26 eV) during its movement between two optimal sites indicates that clustering can be prevented if many molecules of CO are adsorbed on the CuCl ML. For HF, the value of the DEB (0.082 eV) implies that the adsorption phenomenon may happen quite easily upon the CuCl ML. The transfer of charge according to Bader charge analysis and the variation in the work function depend only on the properties of the elements involved, *i.e.*, their nature, rather than the local binding environment. The work function and band-gap energy variation of the CuCl ML (before and after adsorption) show high sensitivity and selectivity of CO and HF binding with the CuCl monolayer. HF molecules give a more rapid recovery time of 1.09 × 10^−7^ s compared to that of CO molecules at a room temperature (RT) of 300 K, which indicates that the necessary adsorption and reusability of the CuCl ML for HF can be accomplished effectively at RT. Significant changes in the conductivity are observed due to the CO adsorption at various temperatures, as compared to adsorption of HF, which suggests the possibility of a modification in the conductivity of the CuCl ML.

## Abstract 2

Using a first-principles approach, the adsorption characteristics of CO and HF on a CuCl monolayer are studied with Grimme-scheme DFT-D2 for accurate description of the long-range (van der Waals) interactions.

## Graphical abstract

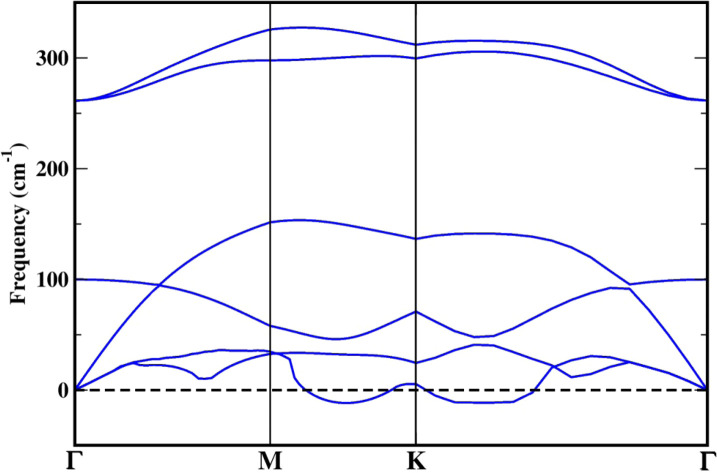

## 1. Introduction

Quality and quantity control of air and toxic gases, respectively, and their monitoring in outdoor as well as indoor environments, like factories, workplaces, laboratories and various industrial settings, have become a serious matter of concern as a result of large emissions of harmful and toxic gases. CO and HF are present in the atmosphere as colorless gases and are produced by partial combustion of organic molecules and in industrial chemical plants, respectively. Continuous and long exposure to these toxic gases can cause serious environmental and health issues.^1,2^ Their exposure can result in strong irritation in the respiratory tract, eye redness, gastrointestinal diseases, rhinorrhea, neurological effects, cough, headache, dermatalgia and other symptoms. Any leakage of these hazardous gases can cause explosions, leading to various kinds of destruction. So, a great deal of work has been carried out for the precise analysis of these gases.^3^

Gas sensors are devices using gas sensing materials for the determination of the concentration and composition of gases in the surrounding area. Therefore, gas sensors are not only used in industry, but also in the field of biomedicine, where they are utilized to investigate exhaled gases to identify different kinds of illness.^4^ Therefore, gas sensing technology needs to show high performance as demanded for national safety and by different industries.^5^ In the gas-detection mechanism, the adsorbed gas molecules can donate or accept electrons from the substrate, resulting in a variation in electrical resistance. So, in this way the presence of gas molecules is detected by the gas sensor. Usually, there are some basic criteria and performance parameters for gas sensors: (a) high sensitivity; (b) high selectivity; (c) stability in performance; (d) fast response; (e) low working temperature and (f) low power consumption. Conventional semiconductor gas sensing technologies, using SnO_2_, TiO_2_, ZnO, and Cu_2_O (semiconducting thin films), are widely studied and employed practically.^6–8^ However, such gas sensors consisting of metal oxides need high temperatures for their operation, some working at temperatures higher than 150 °C, to enhance the chemical reactivity of the gas with the sensing material. As a result of this, the energy consumption increases, therefore reducing their suitability under daily environmental conditions. Room temperature (RT) sensors are lower-cost because they do not need heat for their operation.

Recently, with the progress in low-dimensional semiconductors, 2D materials have attracted much consideration. By using 2D materials, low-power and high-density gas sensors with more sensitivity can be developed. The large surface–volume ratio of 2D materials enables them to have high sensitivity and greater recovery efficiency.^9,10^ They have good conducting and semiconducting features. Surface modifications can also be carried out on these materials due to weak van der Waals forces, which make 2D materials more suitable when compared with 0D and 1D materials. 2D materials can be categorized as: (a) the graphene family;^11^ (b) 2D metal oxides;^12^ (c) transition metal dichalcogenides (TMDCs)^13^ like WS_2_,^14^ WSe_2_,^15^ MoS_2_,^16,17^ and so on; (d) MXenes;^18^ and (5) materials based on a single element, like black phosphorous,^19^ arsenene,^20^ and antimonene.^21^

Cuprous chloride (CuCl) is an ionic semiconductor that has various different applications. It is an essential chemical product in fields like metallurgy, pigments, petrochemicals, and medicine. It is in the class of binary compounds that are tetrahedrally coordinated. These compounds hold huge interest for the research field as well as for understanding basic semiconductor physics. CuCl is a semiconductor with a large band gap^22^ and is studied for its linear and non-linear optical properties.^23^ The fundamental electronic structure of CuCl has been studied by various groups using density functional theory (DFT).^24,25^ CuCl has amazing tunable properties in nanoelectronics, including for gas sensing.^26–29^ CuCl monolayers, having a two-dimensional structure, possess promising properties in gas sensing owing to their high surface–volume ratio. Sun *et al.*,^30^ Kou *et al.*,^31^ Zhu *et al.*,^32^ and Zhang *et al.*^33^ have studied CO adsorption on InN, phosphorene, Janus Te_2_Se and doped graphene monolayers. Recent studies also demonstrated HF and CO adsorption.^34,35^ N. Ahmadian *et al.* discovered an appropriate and sensitive sensor for dimethyl methylphosphonate (DMMP, a nerve agent) on the exterior surface of defect-containing semiconducting (10,0) single-wall carbon nanotubes (SWCNTs), by using first-principles van der Waals density functional (vdW-DF) calculations.^36^ M. D. Ganji *et al.* studied the adsorption of formaldehyde (H_2_CO) on graphene, hexagonal silicon carbide (h-SiC) and hexagonal aluminum nitride (h-AlN) monolayer sheets for application as gas sensors.^37^ M. D. Ganji *et al.* also used DFT simulations to study the adsorption characteristics of acetone on zigzag single-walled boron nitride nanotubes (BNNTs).^38^ T. Banibairami *et al.* used vdW-DF to assess the adsorption of the gas molecule phosgene (COCl_2_) on a hexagonal aluminum nitride (h-AlN) nanosheet.^39^ Here, in our theoretical research, the gas sensing properties of CuCl MLs for CO and HF are studied in detail, which has not been presented before.

Using a first-principles study based on DFT, the adsorption of CO and HF gaseous molecules on CuCl MLs is studied. Correction in the van der Waals interactions is carried out *via* DFT-D2 and the Hubbard potential (*U*) is introduced to take into account the electronic interactions for the strongly correlated orbitals. Geometry optimization is carried out to get highly stable configurations.

## 2. Computational detail

For the structural optimization and investigation of electronic properties, an *ab initio* code, namely the Quantum Espresso (QE) package,^40^ is used. The generalized gradient approximation (GGA) in the Perdew–Burke–Ernzerhof (PBE) functional^41^ is employed for the plane-wave basis set in order to relax the structures, and ultra-soft pseudo-potentials^42^ were adopted for expressing valence electrons and ionic core interactions. In addition, Grimme-scheme DFT-D2 was utilized^43^ for accurate description of the long-range (van der Waals) interactions between the monolayer and gas molecules. The DFT+U method is used for considering the Coulomb repulsion interaction of electrons in the Cu:3d and Cl:3p orbitals, and it is also used for the correction of the self-interaction error (SIE), which is involved in the s, p, d and f states.^44^ So, for this purpose, the addition of the Hubbard parameter (*U*), is carried out in the PBE functional.^45,46^ The *U* parameters were adopted according to Monteiro *et al.*^47^ as *U* (Cu:3d) = 7.0 eV and *U* (Cl:3p) = 7.0 eV, which are attained using the ACBN0 method.^48^

A CuCl monolayer with a 3 × 3 supercell is used, comprising 18 atoms of Cu and Cl (nine for each). To keep apart the adjacent periodic images of the 2D layer of the simulated supercell geometry, a vacuum of 20 Å is set. Structural relaxation is carried out unless the force on an individual atom is less than 0.005 eV Å^−1^. A kinetic-energy cutoff for the wave function of 650 eV and an energy convergence value of 10^−8^ eV is used. TheMonkhorst–Pack scheme^49^ is used for first Brillouin-zone representation with a 4 × 4 × 1 *k*-mesh. For the accuracy of the electronic calculations, a dense *k*-mesh of 12 × 12 × 1 is used for Brillouin-zone sampling. Bader charge analysis^50^ is employed to investigate the charge that is transferred between the CuCl ML and gas molecule. To evaluate the strength of the adsorption phenomenon of the gas molecule on the CuCl ML, the following relation is used:

1 *E*_ads_ = *E*_total_ − (*E*_CuCl_ + *E*_gas molecule_)

where *E*_ads_ is the adsorption energy, *E*_total_ is the total energy of the combined system (a gas molecule adsorbed on the CuCl ML), *E*_CuCl_ is the energy of the pristine CuCl ML and *E*_gas molecule_ is the energy of the gas molecule.

In order to locate the lowest energy pathway between the initial and final coordinates during the adsorption phenomenon, the climbing image nudged elastic band (CI-NEB) method is adopted. For this purpose, a transition state search (TSS) is carried out. The diffusion energy barrier (DEB) is calculated as:

2 *E*_DEB_ = *E*_TS_ − *E*_IS_

where *E*_TS_ is the transition-state energy and *E*_IS_ is the initial-state energy.

The conductivity of the CuCl ML also varies due to the adsorption phenomenon of the gaseous molecules, which is determined by the equation given below.3
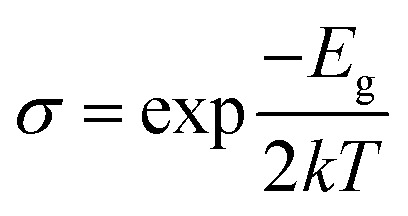
where *σ* denotes the electrical conductivity, *T* is the temperature, *E*_g_ represents the band-gap energy and *k* denotes the Boltzmann constant.

For the determination of the recovery time of the gaseous molecules, the following relation is used:4
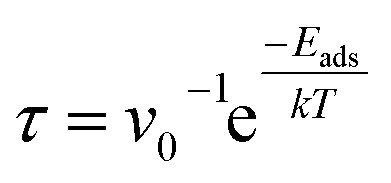
where *E*_ads_ is the adsorption energy, *v*_0_ (=10^12^ s^−1^)^51^ is the attempt frequency, *T* is the temperature and *k* (=8.617 × 10^−5^ eV K^−1^) is the Boltzmann constant.

The work function is the least amount of energy needed for electron ejection from the Fermi level to infinity, for the adsorbed gas molecules. The work function is calculated using the following equation:

5 *ϕ* = *V*(*ϕ*) − *E*_Fermi_

where *ϕ* denotes the work function, *V*(*ϕ*) is the electrostatic potential and *E*_Fermi_ is the Fermi energy of the CuCl ML.

## 3. Results and discussion

**Fig. 1:**
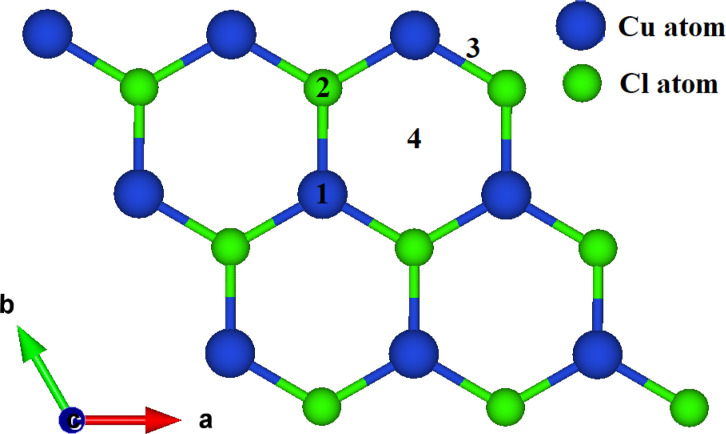
CuCl ML, with different adsorption sites.

For the optimized binding configuration, four adsorption sites on the relaxed pristine CuCl ML are considered for the adsorption of CO and HF. Sites 1 and 2 represent the tops of the Cu atom and Cl atom, respectively. The top of the Cu–Cl bond is site 3 and site 4 represents the CuCl hexagon center, as shown in Fig. 1.

**Fig. 2:**
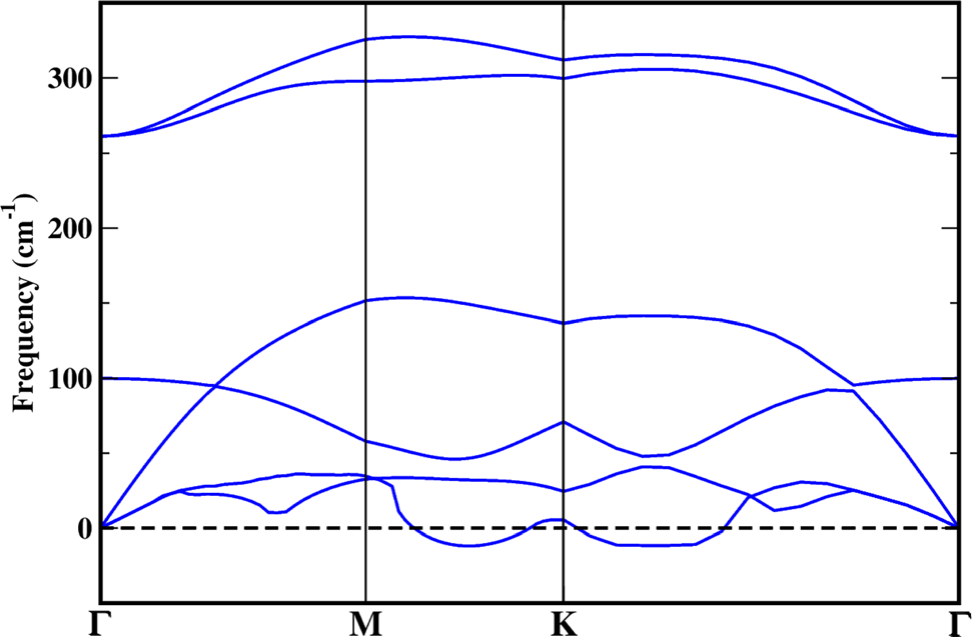
Phonon dispersion of the CuCl monolayer.

The dynamic stability of the CuCl ML structure has been assessed by observing the lattice vibrations with the phonon dispersion plot, as shown in Fig. 2. Only one mode of the phonon dispersion plot of the CuCl ML exhibits a slight imaginary frequency, in the range of about 11 cm^−1^ within the *M* → *Γ* high symmetry *k*-point path, in accordance with a previous theoretical study on the CuCl ML.^27^ This further suggests that it can be stabilized on supporting materials because suitable substrates have always been used in the synthesis of such monolayer-based structures.^52^ As an example, the synthesis of a thin layer of copper iodide is carried out on top of a Cu (111) surface.^53^ Moreover, an ionic magnesium chloride monolayer structure has been produced on Pd, Pt, and Rh metal surfaces.^54^ As a result, we anticipate that such an ML structure may also be achievable through experimentation.

**Fig. 3:**
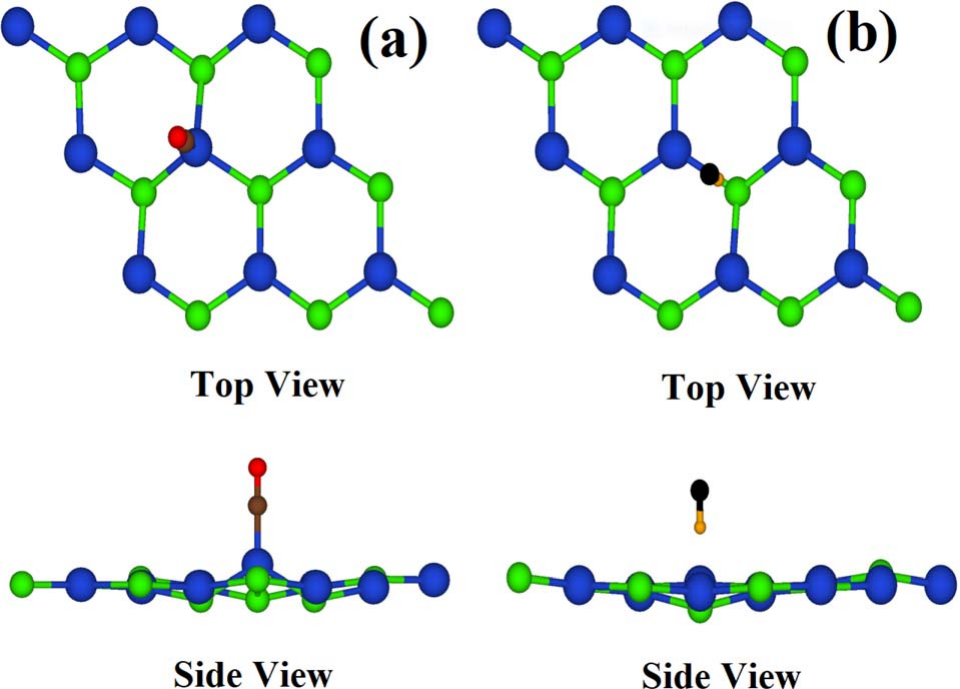
Top and side views of the optimized configurations for adsorption of (a) CO and (b) HF on the CuCl ML. (Colour code: brown, carbon; red, oxygen; yellow, hydrogen; black, fluorine).

The adsorption energy of CO and HF on the CuCl ML is calculated by using eqn (1). According to our calculations, site 1 (Cu atom top) gives the maximum value of adsorption energy for CO, and for HF, site 2 (Cl atom top) is the maximum adsorption energy site. Each gaseous molecule is diatomic, so all the feasible orientations are considered, *i.e.*, they have two possible orientations at the respective sites of maximum adsorption energy, with either of the atoms in the gaseous molecule pointing towards Cu or Cl. The proposed sites result in the configurations shown in Fig. 3, after many minimization steps in each optimization.

The absorption sites with their energies for CO and HF molecules are given in Table 1. It is observed that the adsorption results in the structural deformation of the CuCl ML (Fig. 3). Both the molecules show a vertical alignment with respect to the CuCl ML. In the case of CO, the C atom points towards Cu atom (Fig. 3a). For the HF molecule, the H atom points towards Cl atom (Fig. 3b). It is found that CO gas molecules show chemical adsorption to the CuCl ML, with an adsorption energy value of −1.80 eV. So, it is concluded that the CuCl ML shows a very good adsorption performance for CO gas molecules in comparison with that of other substrates in previous studies, like InN (−0.223 eV), phosphorene (0.325 eV), Janus Te_2_Se (−0.171 eV), graphene (P-graphene, −0.12 eV; B-graphene, −0.14 eV; N-graphene, −0.1 eV), and monolayer ZnS (−0.96 eV), as well as pristine hBN (0.21 eV) and Ti-doped hBN (1.66 eV).^30–35^ Meanwhile, the HF molecule shows physical adsorption to the CuCl ML, with a smaller physical adsorption energy of −0.31 eV. Even so, the CuCl monolayer shows a better adsorption energy for HF molecules as compared to that of Ti-doped hBN, 0.27 eV.^35^ The adsorption of CO and HF molecules is exothermic, as indicated by the negative values of the adsorption energy. Optimal distances of 1.79 Å and 2.09 Å from the CuCl ML are observed for the CO and HF gas molecules, respectively.

**Table 1:** Adsorption energies (*E*_ads_ (eV)), minimum adsorption heights (*h*), charge transfers (*Q*), and diffusion energy barriers (DEBs) for the CuCl ML

Molecule	Site	*h* (Å)	*E*_ads_ (eV)	*Q* (e)	DEB
CO	Cu (site 1)	1.79	−1.80	0.03	1.26
	Cl (site 2)	3.35	−0.12	0.02	—
	Bridge (site 3)	1.74	−1.78	0.01	—
	Centre (site 4)	2.37	−0.30	0.01	—
HF	Cu (site 1)	2.21	−0.22	−0.03	—
	Cl (site 2)	2.09	−0.31	−0.04	0.08
	Bridge (site 3)	2.10	−0.28	−0.021	—
	Centre (site 4)	1.60	−0.26	−0.02	—

**Fig. 4:**
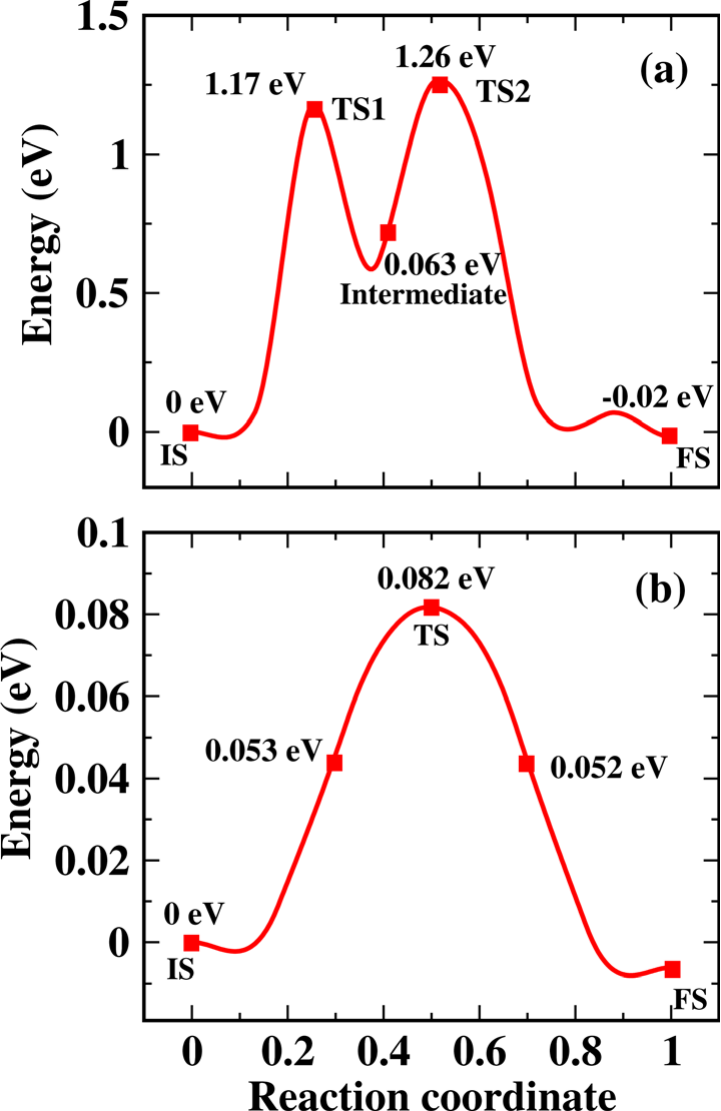
Diffusion energy barriers and diffusion pathways of (a) CO and (b) HF. IS is the initial state, FS is the final state and TS is a transition state.

The transition-state search is carried out for more understanding of the optimized configuration of CO and HF. For this purpose, two adsorption sites are selected, as shown in Fig. 4. The nudged elastic band (NEB) model is used to obtain the minimum energy path between the reactants and products. The paths with their energies indicate that for the adsorption of CO and HF, the lowest-energy site is chosen, which provides proof that the calculated adsorption energy is accurate. Two adsorption states, TS1 and TS2, are noticed in the case of the CO-CuCl system, and for the calculation of the diffusion energy barrier (DEB), transition state TS2 is considered. An intermediate state is also observed in this case, which is a short-lived and unstable state during the adsorption of the gas molecule, between the initial site and final optimal state. Here, in the case of CO-CuCl, the gas molecule CO is briefly adsorbed by the Cl atom on the way from TS1 to TS2. By using eqn (2), the DEB is calculated. The values of the DEB for CO and HF on the surface of the CuCl ML are found to be 1.26 eV and 0.08 eV, respectively. In the case of CO, the greater DEB value indicates that clustering may be avoided when many molecules of CO are introduced on the CuCl ML. However, it is concluded that the binding phenomenon may occur more easily in case of HF upon the CuCl ML, as suggested by its DEB value, even in the absence of external energy.

**Fig. 5:**
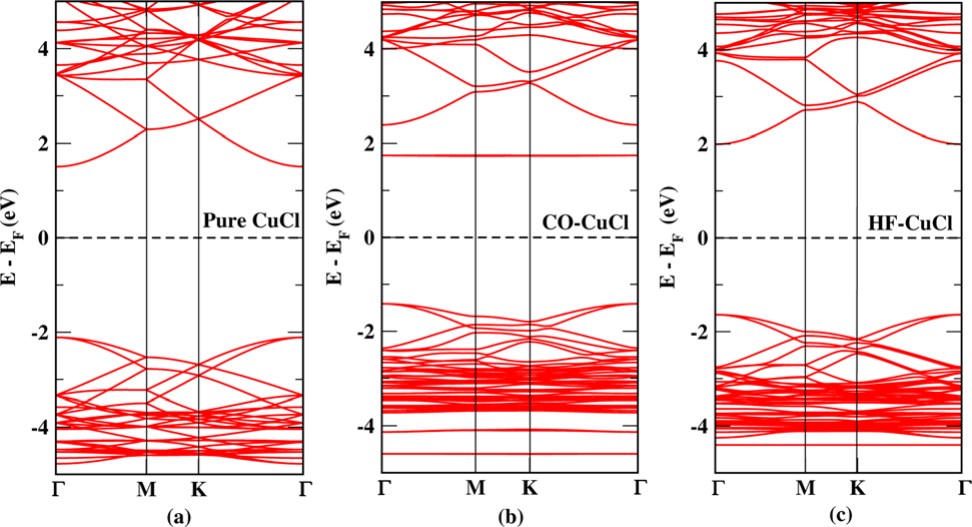
Band structures of (a) the CuCl ML, (b) CO adsorbed on CuCl and (c) HF adsorbed on CuCl.

The calculation of electronic properties is carried out for understanding the interaction of the CuCl ML with the gas molecules. Fig. 5a shows the band structure for the pristine CuCl ML with a band gap of 3.66 eV, which is in accordance with that from previous DFT studies (3.66 eV) and much greater than the 1.1 eV obtained with GGA-PBE.^27^ Both the adsorbed systems, CO and HF on the CuCl ML, show a small increase in the number of conduction bands as compared to the pristine CuCl system (Fig. 5(b) and (c)).

It is also observed that the band-gap energy decreased to 3.14 eV in the case of the CO-CuCl system and 3.56 eV for the HF-CuCl system, as given in Table 2.

**Table 2:** Band-gap energies (*E*_g_) and work functions (*ϕ*) of CuCl and with gas-molecule adsorption

System	*E*_g_ (eV)	*ϕ* (eV)
CuCl ML	3.66	4.26
CO-CuCl	3.14	4.48
HF-CuCl	3.56	4.74

**Fig. 6:**
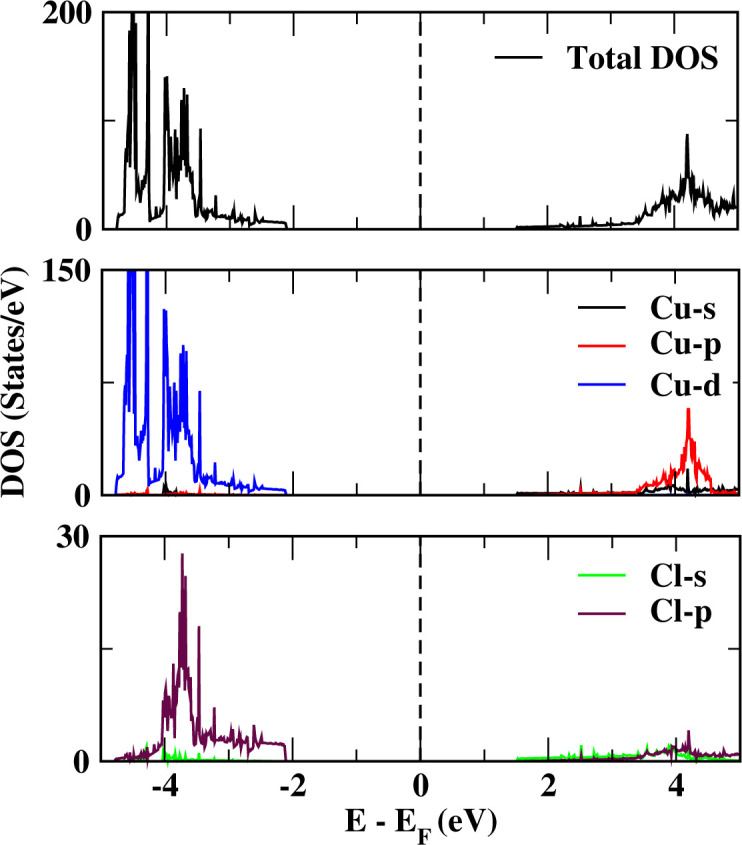
Projected density of states (PDOS) of the pristine CuCl.

**Fig. 7:**
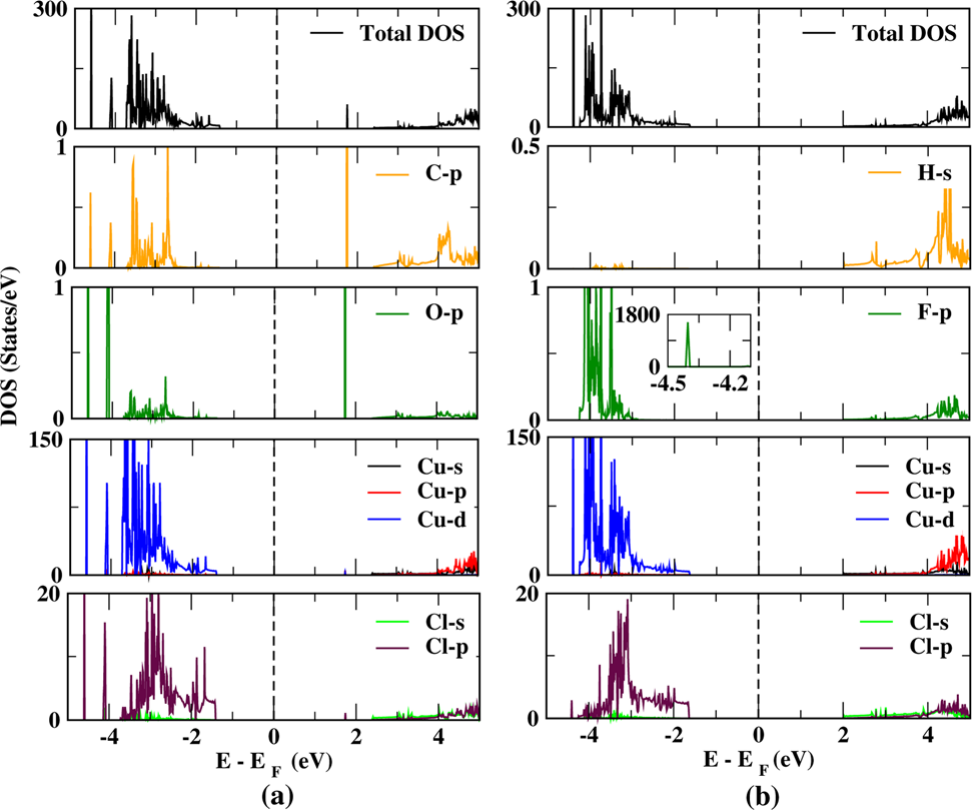
Projected density of states (PDOS) the (a) CO-CuCl and (b) HF-CuCl systems.

According to our calculations, the CuCl ML has a nonmagnetic nature with and without the adsorption of CO and HF. To analyze the contribution of the CO and HF adsorbed on the CuCl ML and the newly appearing band lines, the total density of states (TDOS) and projected density of states (PDOS) are plotted (Fig. 7a and b) in comparison to those of the pristine CuCl ML (Fig. 6). New peaks are observed at about 1.75 eV (due to C 2p and O 2p orbitals) in case of CO-CuCl, whereas a peak is observed at about −4.4 eV (due to F 2p orbitals) for the HF-CuCl system. Moreover, the three systems, *i.e.*, pristine CuCl, CO-CuCl and HF-CuCl, have filled valence bands due to the bonding states (Cu d and Cl p orbitals). In contrast, the conduction-band bottom is made from the unoccupied orbitals (Cu s, d and Cl p) for the pristine CuCl system.

Bader charge analysis gives the charge transfer between the CuCl monolayer and gaseous molecules. It is calculated that the charge transfer between the HF and CuCl monolayer is −0.04*e*, which is a bit greater than that in previous studies,^35^ and similarly we observed a high charge transfer of 0.03*e* for the CO/CuCl ML as compared to that in previous studies.^30,32–35^ It is observed from the calculations that the charge transfer is independent of the Hubbard parameter, *i.e.*, the local binding environments, and only relies upon the properties of the adsorbed gas and the nature of the monolayer, which is compatible with the findings of previous studies.^55^

**Fig. 8:**
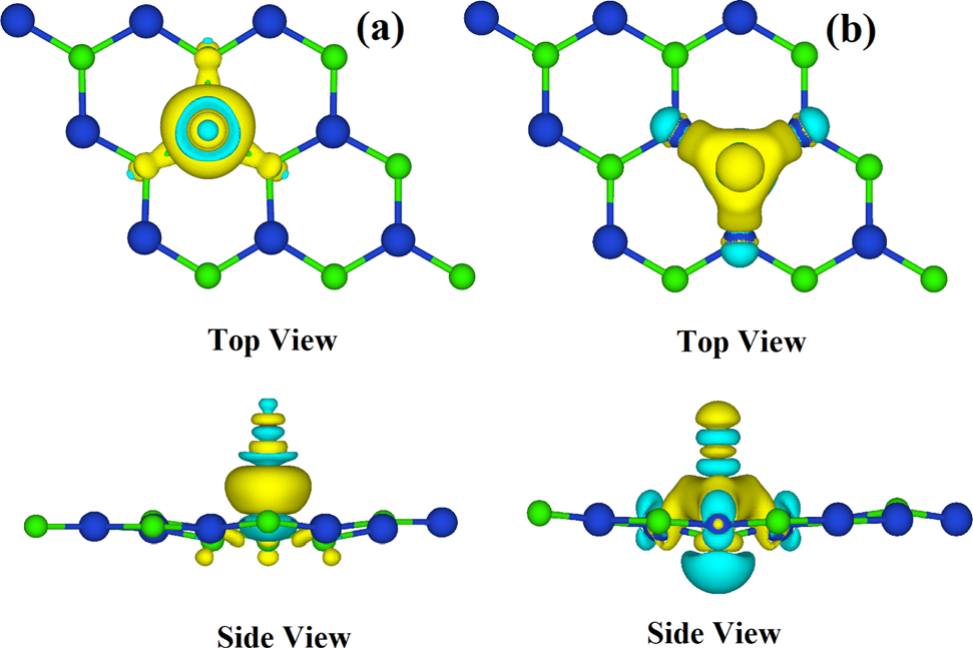
Charge density difference of (a) CO-CuCl and (b) HF-CuCl.

Furthermore, the calculation of electronic charge density difference is carried out to provide more insight into the charge transfer for the CO-CuCl and HF-CuCl systems, as displayed in Fig. 8a and b, respectively. The cyan colour shows charge depletion, whereas the yellow colour shows charge accumulation at an isovalue of 0.97 meV A^−3^. The CO gas molecule behaves as a charge donor due to charge accumulation on the CuCl ML, whereas HF accepts electrons from CuCl and behaves as a charge acceptor. These results are in accordance with the Bader charge analysis. The shifting of charge due to exposure to gases has a major influence on the resistance. The reduction of charge carriers takes place due to charge depletion in the monolayer and increases its resistance. However, reduction in the resistance takes place as a result of accumulation of charges in the monolayer, because accumulation increases the charge carriers.

**Fig. 9:**
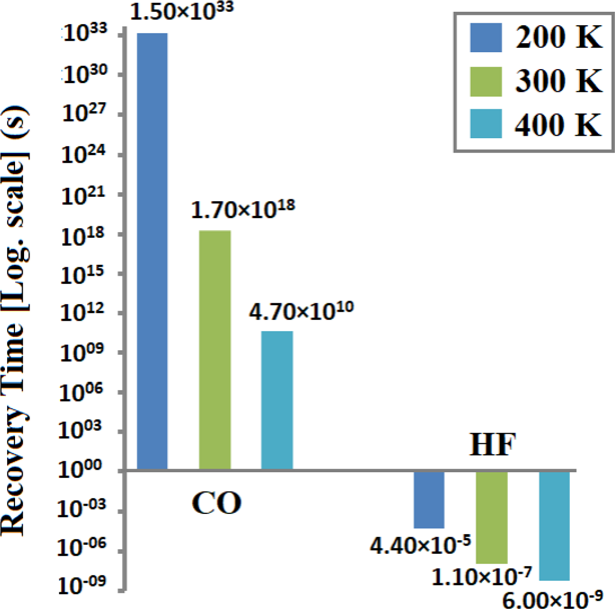
Recovery times for CO and HF adsorbed on the CuCl monolayer.

Determination of the recovery time is required to find the reusability of the gas sensors. It is calculated for CO and HF at various temperatures using eqn (4). Fig. 9 shows the plot of the recovery time. It is observed that at RT, the value of the recovery time of CO on CuCl ML is 1.69 × 10^18^ s, while HF molecules show a more rapid recovery time of 1.09 × 10^−7^ s, which is much less than that found in previous studies on HF (4.68 min).^34^ This indicates that we can achieve active sensing with a fast recovery time for HF at RT (300 K).^56,57^ Further calculations are carried out to determine the changes with various temperatures. At 200 K, CO gave a recovery time of 1.5 × 10^33^ s and for HF its value is 4.4 × 10^−5^ s. Moreover, at a high temperature of 400 K, the recovery time of CO is 4.70 × 10^10^ s and for HF its value is 6.0 × 10^−9^ s, as shown in Fig. 9. Lowering the temperature gives a higher recovery time, whereas an elevation in the temperature reduces its value to an acceptable range. For CO molecules, the recovery time can be reduced by increasing the temperature or by using UV radiation (attempt frequency *v*_0_ = 10^15^ s^−1^). This observation suggests that the reusability and required sensing of HF on the CuCl ML may be achieved efficiently at RT.

Moreover, the variation in the carrier mobility also brings changes in the conductivity of the CuCl ML due to gas adsorption, which is determined using eqn (3). The conductivity shows a direct relation to the exponential of band gap. Any variation in the band-gap width alters the conductivity. Our calculations show a band-gap energy difference of 0.52 eV for CO adsorption and 0.1 eV for HF adsorption on the CuCl ML. The energy change in the gap before and after adsorption shows high selectivity for CO and HF with the CuCl monolayer. Although there is less observed band-gap energy variation, its exponential value leads to a notable change in the conductivity at various temperatures. This observation shows the possibility of a modification in the conductivity of the CuCl ML due to CO and HF adsorption. This also suggest that CO and HF can be distinguished through the energy-gap variation measurement.

**Fig. 10:**
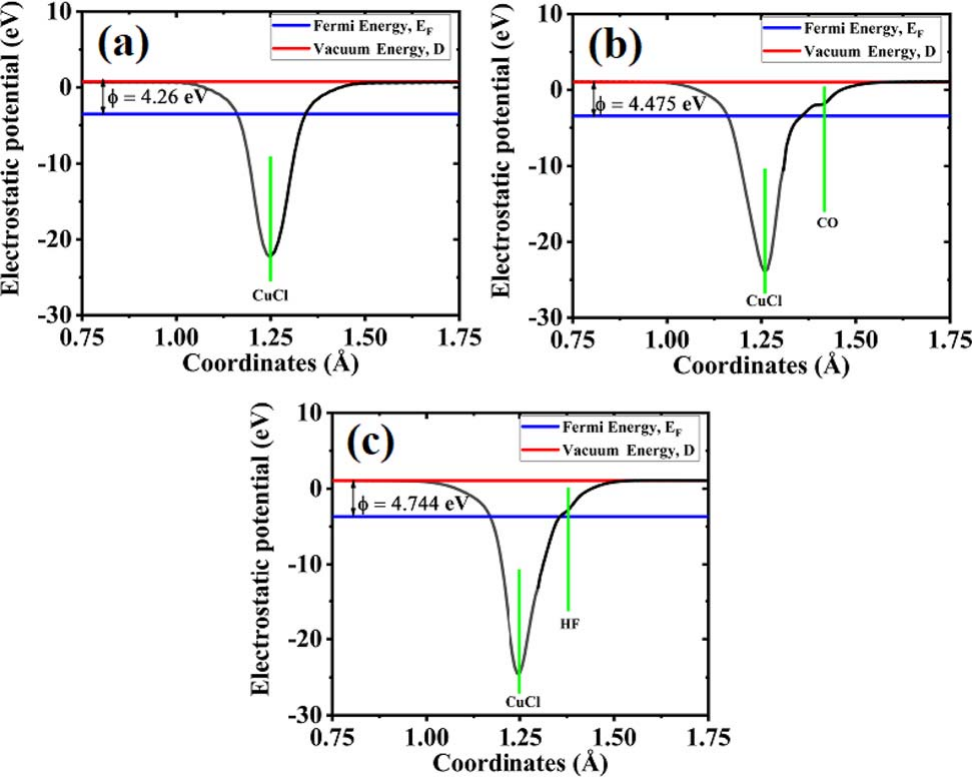
Work functions for the (a) CuCl ML, (b) CO-CuCl and (c) HF-CuCl systems.

The work function is the difference between the electrostatic potential in a vacuum and the Fermi level. It is calculated using eqn (5). The work function for the pristine CuCl ML is 4.26 eV, whereas after the adsorption of gas molecules on the CuCl ML, the value of the work function is increased to 4.475 eV for CO molecules and 4.74 eV for HF, as shown in Fig. 10a–c. The work function is related to the conductivity, because it changes due to variations in the charge concentration.^58^ Similar to the Bader charge analysis discussed earlier, the local binding environment also does not affect the work-function variation, but its dependence is on the nature of the elements involved. On the whole, the work-function variation confirms a strong interaction of CO and HF with the CuCl ML, which indicates the sensitivity of the CuCl ML to CO and HF molecules.

## 4. Conclusion

Our DFT+U study suggests that the CuCl monolayer shows favorable chemical adsorption for the CO molecule and physical adsorption for the HF gas molecule. CO possesses a quite strong adsorption energy of −1.80 eV, whereas HF has a −0.31 eV adsorption energy. For the CO gas molecule, the large value of the diffusion energy barrier (DEB) during its movement between two optimal sites indicates that clustering can be prevented if many molecules of CO are adsorbed on the CuCl ML. For HF, the value of the DEB implies that the adsorption phenomenon may happen quite easily upon the CuCl ML. The transfer of charge according to the Bader charge analysis and the variation in the work function depend only on the properties of the elements involved, *i.e.*, its nature, rather than the local binding environment. The difference in the work function of the CuCl ML (before and after adsorption) verifies the presence of a strong interaction and the high sensitivity of the CuCl monolayer to CO and HF gas molecules. This change also suggests that both gases, CO and HF, can be distinguished through the work-function measurement. HF molecules show a more rapid recovery time than CO molecules at room temperature, which indicates that the necessary adsorption and reusability of CuCl ML for HF can be accomplished effectively at 300 K. Significant changes in the conductivity are observed due to the CO adsorption at various temperatures as compared to HF, suggesting the possibility of a modification in the conductivity of the CuCl ML and showing high selectivity of the CuCl ML for CO and HF. These findings show that the CuCl monolayer could be a promising candidate for CO and HF adsorption. This research study is expected to give a better insight into the sensing properties of the CuCl ML and the chances of tuning such properties by effective means in future studies.

## Conflicts of interest

The authors declare that they have no known competing financial interests or personal relationships that could have appeared to influence the work reported in this paper.

## Supplementary Material
